# O-RADS MRI scoring system: key points for correct application in inexperienced hands

**DOI:** 10.1186/s13244-024-01670-3

**Published:** 2024-04-12

**Authors:** Lledó Cabedo, Carmen Sebastià, Meritxell Munmany, Pere Fusté, Lydia Gaba, Adela Saco, Adela Rodriguez, Blanca Paño, Carlos Nicolau

**Affiliations:** 1 Department of Radiology, Hospital Clínic de Barcelona, C/Villarroel, Barcelona, 170 08036 Spain; 2 Department of Gynaecology and Obstetrics, Hospital Clínic de Barcelona, C/Villarroel, Barcelona, 170 08036 Spain; 3 Department of Oncology, Hospital Clínic de Barcelona, C/Villarroel, Barcelona, 170 08036 Spain; 4 Department of Pathology, Hospital Clínic de Barcelona, C/Villarroel, Barcelona, 170 08036 Spain; 5 August Pi i Sunyer Biomedical Research Institute (IDIBAPS), Barcelona, Spain

**Keywords:** Cancer, MRI, Ovary

## Abstract

**Objectives:**

To evaluate the efficacy of the O-RADS MRI criteria in the stratification of risk of malignancy of solid or sonographically indeterminate ovarian masses and assess the interobserver agreement of this classification between experienced and inexperienced radiologists.

**Methods:**

This single-centre retrospective study included patients from 2019 to 2022 with sonographically indeterminate or solid ovarian masses who underwent MRI with a specific protocol for characterisation according to O-RADS MRI specifications. Each study was evaluated using O-RADS lexicon by two radiologists, one with 17 years of experience in gynaecological radiology and another with 4 years of experience in general radiology. Findings were classified as benign, borderline, or malignant according to histology or stability over time. Diagnostic performance and interobserver agreement were assessed.

**Results:**

A total of 183 patients with US indeterminate or solid adnexal masses were included. Fifty-seven (31%) did not have ovarian masses, classified as O-RADS 1. The diagnostic performance for scores 2–5 was excellent with a sensitivity, specificity, PPV, and NPV of 97.4%, 100%, 96.2%, and 100%, respectively by the experienced radiologist and 96.1%, 92.0%, 93.9%, and 94.8% by the inexperienced radiologist. Interobserver concordance was very high (Kappa index 0.92). Almost all the misclassified cases were due to misinterpretation of the classification similar to reports in the literature.

**Conclusion:**

The diagnostic performance of O-RADS MRI determined by either experienced or inexperienced radiologists is excellent, facilitating decision-making with high diagnostic accuracy and high reproducibility. Knowledge of this classification and use of assessment tools could avoid frequent errors due to misinterpretation.

**Critical relevance statement:**

Up to 31% of ovarian masses are considered indeterminate by transvaginal US and 32% of solid lesions considered malignant by transvaginal US are benign. The O-RADs MRI accurately classifies these masses, even when used by inexperienced radiologists, thereby avoiding incorrect surgical approaches.

**Key points:**

• O-RADS MRI accurately classifies indeterminate and solid ovarian masses by ultrasound.

• There is excellent interobserver agreement between experienced and non-experienced radiologists.

• O-RADS MRI is a helpful tool to assess clinical decision-making in ovarian tumours.

**Graphical Abstract:**

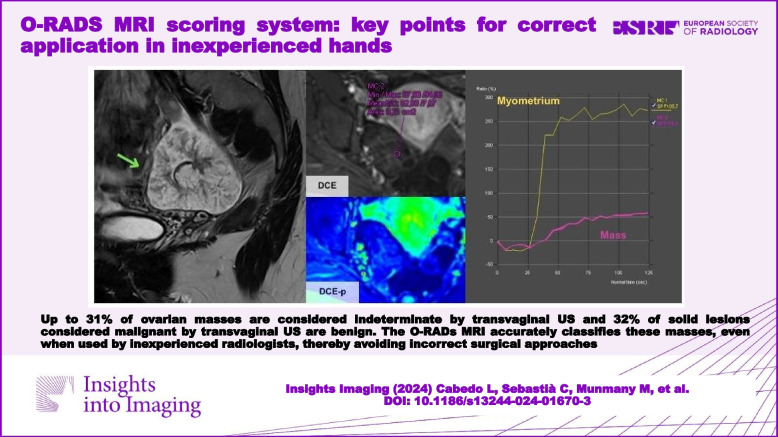

## Introduction

Adnexal masses are a common pathology in women resulting in an important public health problem and a significant workload in hospitals and other healthcare centres [1, 2]. In western countries, 10% of women undergo surgery for adnexal masses, but the majority of these interventions correspond to benign tumours and less than 15% are performed because of the presence of ovarian cancer [3]. In cystic masses, the prevalence of malignancy after surgery is only 3.6% [4]. However, ovarian cancer remains the first cause of mortality due to gynaecological cancer in developed countries [3].

Gynaecological ultrasonography (US) is the first test of choice as most adnexal masses can be accurately categorised as benign or malignant with this tool [5, 6]. However, up to 31% of adnexal masses are found to be indeterminate in the US study, using International Ovarian Tumour Analysis Simple Rules (IOTA-SR) or other US scoring systems [7]. Benign solid ovarian tumours, such as fibromas, can be misclassified by US in up to 32% of cases [8, 9]. Percutaneous biopsy of adnexal lesions is contraindicated because of the high risk of peritoneal seeding in malignant tumours and the poor diagnostic performance of the test [10, 11].

The complexity of managing adnexal masses lies in avoiding underdiagnosis of malignant lesions and overdiagnosis of benign lesions [12]. Treating malignant ovarian masses as benign with conservative surgery done in non-oncologic centres leads to suboptimal cytoreduction and poorer clinical outcomes [13]. On the other hand, treating benign ovarian masses as malignant entails unnecessary aggressive surgical intervention with a higher risk of loss of fertility and greater morbidity [14, 15].

In these cases, magnetic resonance imaging (MRI) has emerged as a problem-solving tool for further assessment and optimisation of patient management following indeterminate US findings or in solid masses which are apparently malignant by US but turn out to be benign [16]. In 2013, Thomassin-Nagara et al. described the ADNEX MR system which established a standardised evaluation of adnexal masses relaying on a specific protocol that showed a sensitivity of 94% and specificity of 97% for the diagnosis of malignant tumours [17]. Subsequently, the ADNEX MR system was validated in different series [18–20]. In 2021, the Ovarian-Adnexal Reporting and Data System (O-RADS) MRI Committee of the American College of Radiology was founded [21]. One year later, the first version of the O-RADS MRI stratification system emerged [22].

MRI analysis of adnexal masses is complex and requires a learning curve. The use of standardised scores homogenises interpretation, provides a structured reporting framework, and can help less experienced readers achieve correct malignancy risk stratification. Therefore, the aim of this study was to assess the diagnostic performance of O-RADS MRI classification in our institution and to evaluate the interobserver agreement between experienced and inexperienced radiologists using these criteria. Additionally, we provide recommendations for avoiding usual beginner mistakes based on our experience.

## Methods

This study was approved by the Research Ethics Committee of our institution (HCB/2023/0234) and was conducted following the principles for medical research involving human subjects, according to the Declaration of Helsinki [23]. Informed consent from the patients was not required as this was a retrospective study based on data obtained from previously performed MRI scans.

### Study population

In this retrospective study, we reviewed the MRIs performed in patients referred to our service for the characterisation of an ovarian mass from January 2019 to December 2022.

A formal calculation of the sample size was not performed, and therefore the sample included the patients attended in our hospital during the study period. To this end, we included all the patients with a suspected ovarian mass considered indeterminate or totally solid by transvaginal US, in whom the complete O-RADS MRI protocol was performed, and who had subsequently undergone surgery or were followed for at least 1 year. The gynaecological US evaluation was performed according to “IOTA-SR” criteria modified by expert clinical opinion.

We excluded all women with clinical suspicion of ovarian torsion or pelvic inflammatory disease at the time the MRI was performed as stated in the O-RADS classification [22]. Also, patients who did not complete the MRI protocol or who were lost to follow-up were excluded. With the current O-RADS classification, according to the American College of Radiology (ACR), these patients should be considered O-RADS 0 [24].

### MRI acquisition and analysis

All the studies were performed in 1.5 or 3 Tesla equipment: Signa Explorer (General Electrics), Magnetom Aera (Siemens) and Magnetom vida (Siemens). Our MRI protocol included all the specific sequences required to apply the O-RADS MRI classification according to the specifications reported in the literature, which are shown in Table 1 [20, 22, 25].

**Table 1:** MRI protocol specifications

	**Siemens 1.5 T**		**GE 1.5 T**		**Siemens 3 T**	
	**Thickness (mm)**	**Intersection Gap (mm)**	**Thickness (mm)**	**Intersection Gap (mm)**	**Thickness (mm)**	**Intersection Gap (mm)**
**Axial T2W**	4	1	4	1	3	0
**Sagittal T2W**	4	1	4	1	3	0
**Coronal T2W**	3	0.3	3	0.3	3	0
**Axial T1W**	5	1	4	0.3	4	0.8
**Axial T1W FS**	5	1	4	0.3	4	0.8
**DWI b1000**	4	0	4	0.4	3	0
**DCE**^a^	3.6	0.7	4	0	3	0.6
**Axial T1W FS + C**	5	1	4	0.3	4	0

*DCE* Dynamic contrast enhancing sequence, *DWI* Diffusion-weighted image

^a^DCE sequences were with a time resolution of 15 s during 3 to 4 min

The medical records of the patients were reviewed. Clinical data such as age, menstruation status, gynaecological symptoms (presence of pelvic pain at the time of MRI acquisition), serum tumour marker values (Ca125 and HE4), and US findings (according to IOTA-SR modified by expert opinion) were recorded. The management strategy (surgery or follow-up) and outcomes after surgery or at 1 year of follow-up were also recorded.

MRI interpretation was performed independently by a junior radiologist (JR) with 4 years of experience in general radiology and a senior radiologist (SR) with 17 years of experience in urogenital and gynaecological radiology, blinded to the patients’ clinical data, US findings and pathological results. The two readers independently characterised each mass according to a standardised lexicon and assigned a score from 1 to 5 for each adnexal mass following the O-RADS MRI [20, 22]. The processing of the DCE, elaboration, and interpretation of the time-intensity curves (TIC) was carried out independently by each radiologist in every study using the Syngo.via software (Siemens). For the elaboration of TIC, both readers placed one region of interest (ROI) within the external myometrium and one ROI within the solid tissue component of the adnexal mass. In cases in which no adnexal mass was present or the pelvic mass did not originate from the adnexa, radiologists were instructed to assign a score of 1. Additionally, they were required to assess the non-adnexal mass and classify it as either suspicious or non-suspicious for malignancy. The JR received interpretation training for the O-RADS MRI prior to the initiation of the study by reviewing all the MRIs performed in our centre for characterisation of pelvic masses for 1 year before starting the study, having access to the histopathological outcome of the operated masses and the clinical outcome of the followed-up ones.

Ovarian masses were classified as benign, borderline, or malignant according to histopathological results, or were considered benign after stability over time at 1 year of follow-up. For statistical analysis, borderline ovarian tumours were considered malignant tumours, as described in the literature [20, 22]. Only true ovarian masses (O-RADS 2–5) were taken into account for the sensitivity and specificity statistical analysis. In patients with more than one adnexal mass, the highest score was considered for the statistical analysis.

### Statistical analysis

Results are shown as absolute and relative frequencies (%), and age is shown as mean and standard deviation. The degree of concordance between the JR and SR was estimated using the Kappa index and the performance of the radiologist and pathologist evaluations was estimated using the sensitivity, specificity and positive (PPV) and negative (NPV) predictive values. All results are expressed with their 95% confidence interval (95% CI). The statistical package SPSS VER 26 (Armonk, NY: IBM Corp) was used for the analyses.

## Results

### Characteristics of the patients and masses

**Fig. 1:**
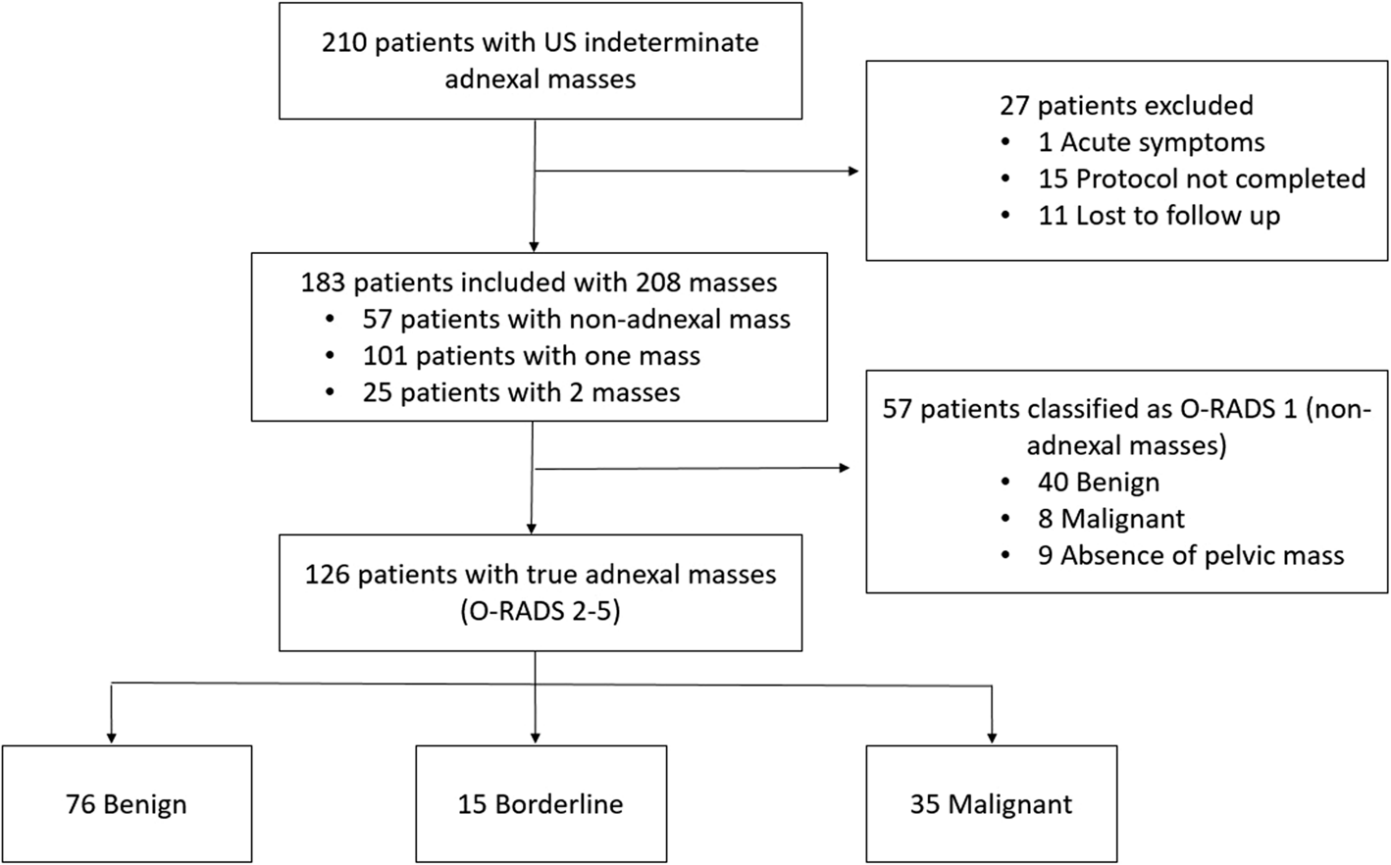
Population flowchart. US, ultrasound. Note that in 25 patients two ovarian masses were found; in these cases, only the mass with the greatest O-RAD score was included. In the 57 patients with O-RADS 1, 40 were benign, 8 malignant, and in 9, no mass was found. Borderline and malignant were considered malignant for statistical purposes

From January 2019 to December 2022, 210 patients were referred to our department to perform an MRI for the characterisation of a US indeterminate adnexal mass. We excluded 27 patients from the study. Fifteen due to the whole MRI protocol for characterisation of an ovarian mass was not completed, 11 were lost to follow-up, and one who presented acute pelvic pain at the time of MRI acquisition (Fig. 1).

A total of 183 women (mean age 51; standard deviation 16) with 208 US indeterminate or solid adnexal masses were included in the study. Twenty-five patients (13,7%) had two adnexal masses, and in these cases, only the mass with the highest score was considered for the statistical analysis. Thus, the final number of masses analysed was 183. The clinical characteristics of the patients are summarised in Table 2. All the patients in the study were discussed at the oncologic gynaecology multidisciplinary committee of our hospital, as is the standard procedure for all patients with indeterminate or suspicious adnexal masses in our centre.

**Table 2:** Characteristics of the study population and masses

	**nº**	**%**
**Nº patients**	183	NA
*Nº patients with non-adnexal lesions*	57	31
*Nº patients with true adnexal lesions*	126	69
**Age of patients mean (SD)**	51.09 (15.79)	NA
**Size of lesions in cm mean (SD)**	7.69 (6.71)	NA
**Menstruation status**		
*Premenopausal*	97	53.00
*Postmenopausal*	86	46.99
**Nº of adnexal lesions**	126	NA
*1 lesion*	100	79.36
*2 lesions*	26	20.63
**Adnexal lesion types**		
*Cystic without solid component or septa*	28	22.22
*Cystic with thin septa*	17	13.49
*Solid dark-dark lesion*	10	7.94
*Irregular septations or walls*	9	7.14
*Papillary projections*	16	12.69
*Mural nodules*	31	24.6
*Large solid portion (no dark-dark)*	15	11.9
**US characteristics**		
*Indeterminate by SR*	125	68.30
*Applies B features but indeterminate by expert opinion*	37	20.22
*Applies M features and totally solid hypervascularised*	21	11.48
***O-RADS MRI score senior (junior)***		
1	57 (57)	31.14 (31.14)
*2*	40 (41)	21.85 (22.4)
*3*	34 (36)	18.57 (19.67)
*4*	18 (15)	9.83 (8.2)
*5*	34 (34)	18.57 (18.57)
**Management of patients with true adnexal lesions**		
*Pathologic analysis*	79	62.69
*Follow-up*	47	37.30
**Final outcome**		
*Benign*	77	61.11
*Borderline*	17	13.49
*Malignant*	32	25.39

*SD* Standard deviation, *US* Ultrasound, *SR* Simple rules

In relation to the US characteristics, 125 out of 183 masses (68.3%) were classified as indeterminate according to “IOTA-SR”; 37 (20.2%) met criteria for category B although seemed suspicious by the expert gynaecologist’s opinion (classified as indeterminate according to “IOTA-SR modified by expert”), and 21 (11.47%) met criteria for category M but were completely solid and hypervascular on Doppler study (Table 2).

**Fig. 2:**
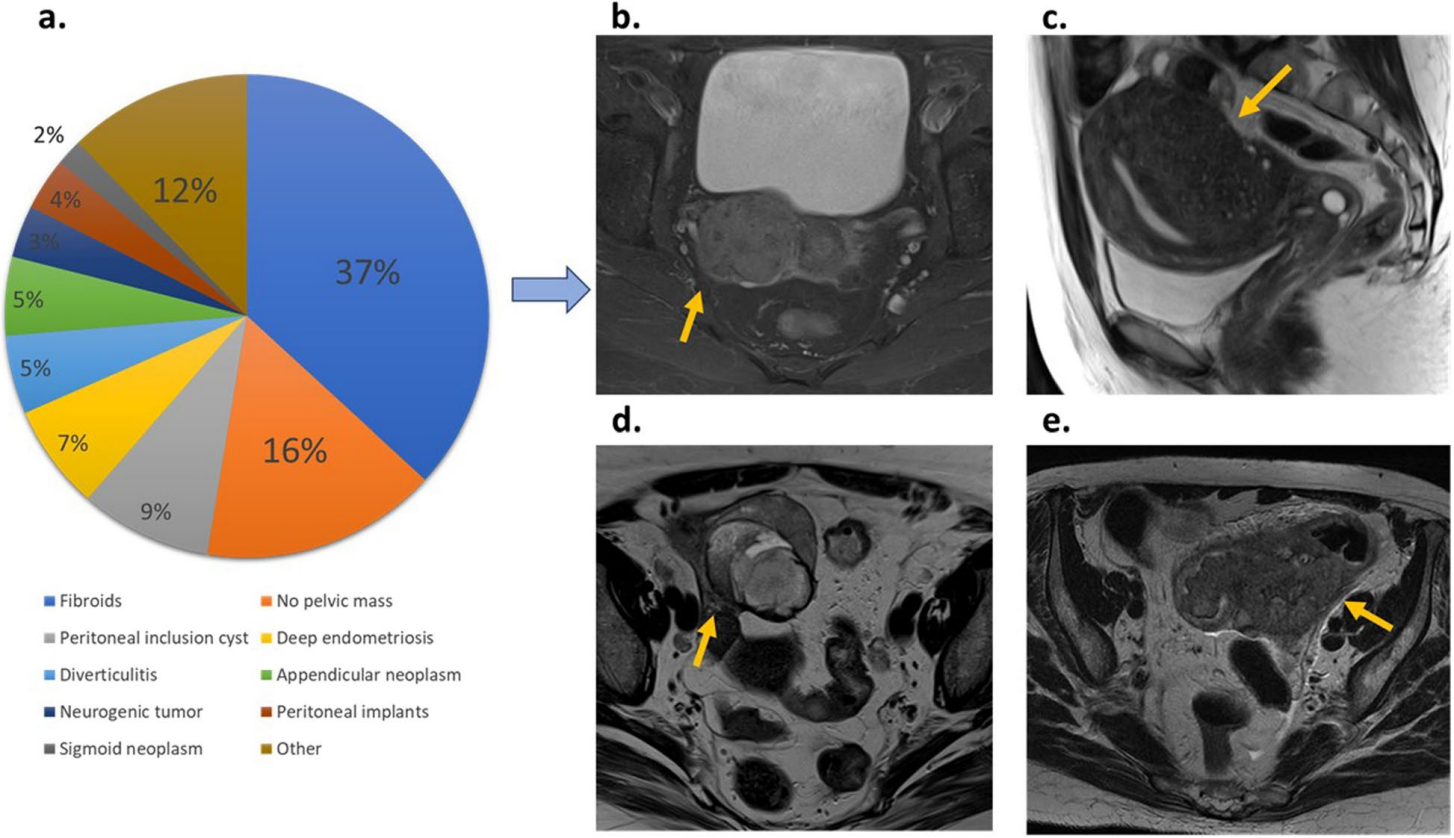
Distribution of non-adnexal masses and examples. **a** O-RADS score 1 distribution. **b** Parametrial fibroid (arrow). **c** Uterine adenomyosis (arrow). **d** Appendiceal gastrointestinal stromal tumour (GIST) (arrow) **e** Sigmoid adenocarcinoma (arrow)

Of the 183 masses, 57 (31%) were extraovarian pelvic masses or physiological ovarian findings classified as O-RADS 1 (Fig. 2). Of these 57 masses, 40 were benign (70.2%), with 21 (36.8%) of these masses corresponding to fibroids. Less frequent extraovarian benign masses included peritoneal inclusion cysts (5, 8.8%), deep endometriosis (4, 7%), and diverticulitis (3, 5.3%). In isolated cases, we found a uterine malformation, an extramedullary haematopoiesis focus, pelvic gross calcifications related to a mesenteric granuloma, endometrial polyp, and a non-gynaecological abscess. In 8 cases (14.1%), malignant extraovarian pelvic masses, such as appendiceal neoplasms (3 patients, 5.3%), neurogenic tumours (2 patients, 3.5%), peritoneal implants (2 patients, 3.5%), or sigmoid neoplasms (1 patient, 1.8%), were found. In 9 (15.7%) patients, no pelvic mass was found.

Of the 126 patients with true adnexal masses, 62 were menopausal. The series comprised 77 benign masses (61.1%), 17 borderline masses (13.5%), and 32 malignant masses (25.4%). Borderline and malignant masses were both considered malignant for the statistical analysis. A total of 80 (63.5%) patients underwent surgery and 46 (36.5%) were followed for at least 1 year. The final diagnoses (histopathology results for the operated adnexal masses and clinical diagnosis after 1 year of follow-up) are summarised in Table 3. None of the 46 patients followed for 1 year presented progression of the disease during the study duration.

**Table 3:** Histopathology results for the operated adnexal masses and clinical diagnosis after 1 year of follow-up

**Pathology (nº)**	**Nº**	**Operated/followed up**	**%**
**Benign (77)**			
Cystic lesion with simple fluid	17	0/17	13.49
Cystic lesion with haemorrhagic content	6	0/6	4.76
Endometrioma	16	2/14	12.70
Fibroma	17	8/9	13.49
Fibrothecoma	2	2/0	1.59
Cystadenofibroma	7	7/0	5.56
Cystadenoma	5	5/0	3.97
Benign germ cell tumour	5	5/0	3.97
Benign Brenner tumour	2	2/0	1.59
**Borderline (16)**			
Serous borderline tumour	12	12/0	9.52
Mucinous borderline tumour	4	4/0	3.17
Seromucinous borderline tumour	1	1/0	0.79
**Malignant (33)**			
Serous carcinoma	7	7/0	5.56
Mucinous carcinoma	1	1/0	0.79
Malignant gem cell tumours	7	7/0	5.56
Clear cell carcinoma	5	5/0	3.97
Endometrial carcinoma	5	5/0	3.97
Anaplastic carcinoma	1	1/0	0.79
Carcinosarcoma	1	1/0	0.79
Endometrial stroma sarcoma	1	1/0	0.79
Follicular lymphoma	1	1/0	0.79
Metastasis	3	3/0	2.38

### Assignment of O-RADS categories and malignancy rates

The JR assigned a score 2 or 3 to 77 of the 126 masses (61.1%). Of these, 4 (5.2%) were finally malignant lesions in the histopathological analysis (a borderline serous tumour, an immature teratoma, an ovarian metastasis from a mucinous appendiceal neoplasm, and a clear cell carcinoma arising from a cystadenofibroma). The SR assigned a score of 2 or 3 to 74 masses (58.7%), and all were benign in the histopathological analysis or remained stable after 1 year of follow-up.

The JR assigned a score 4 or 5 to 49 of the 126 masses (38.9%). Of these, 3 (6.1%) were finally benign lesions (two fibrothecomas and one mucinous cystadenoma mixed with a Brenner tumour). The SR assigned a score 4 or 5 to 52 of the 126 masses (41.2%). Of these, 2 (3.8%) were benign lesions (two fibrothecomas, one with luteinisation).

In the case of the JR, the percentage of malignancy in O-RADS MRI scores 2, 3, 4, and 5 was 1.20%, 10.80%, 93.30%, and 93.80%, respectively. The percentage of malignancy according to O-RADS MRI scores 2, 3, 4, and 5 by the SR was 0%, 1%, 100%, and 94.10%, respectively.

**Fig. 3:**
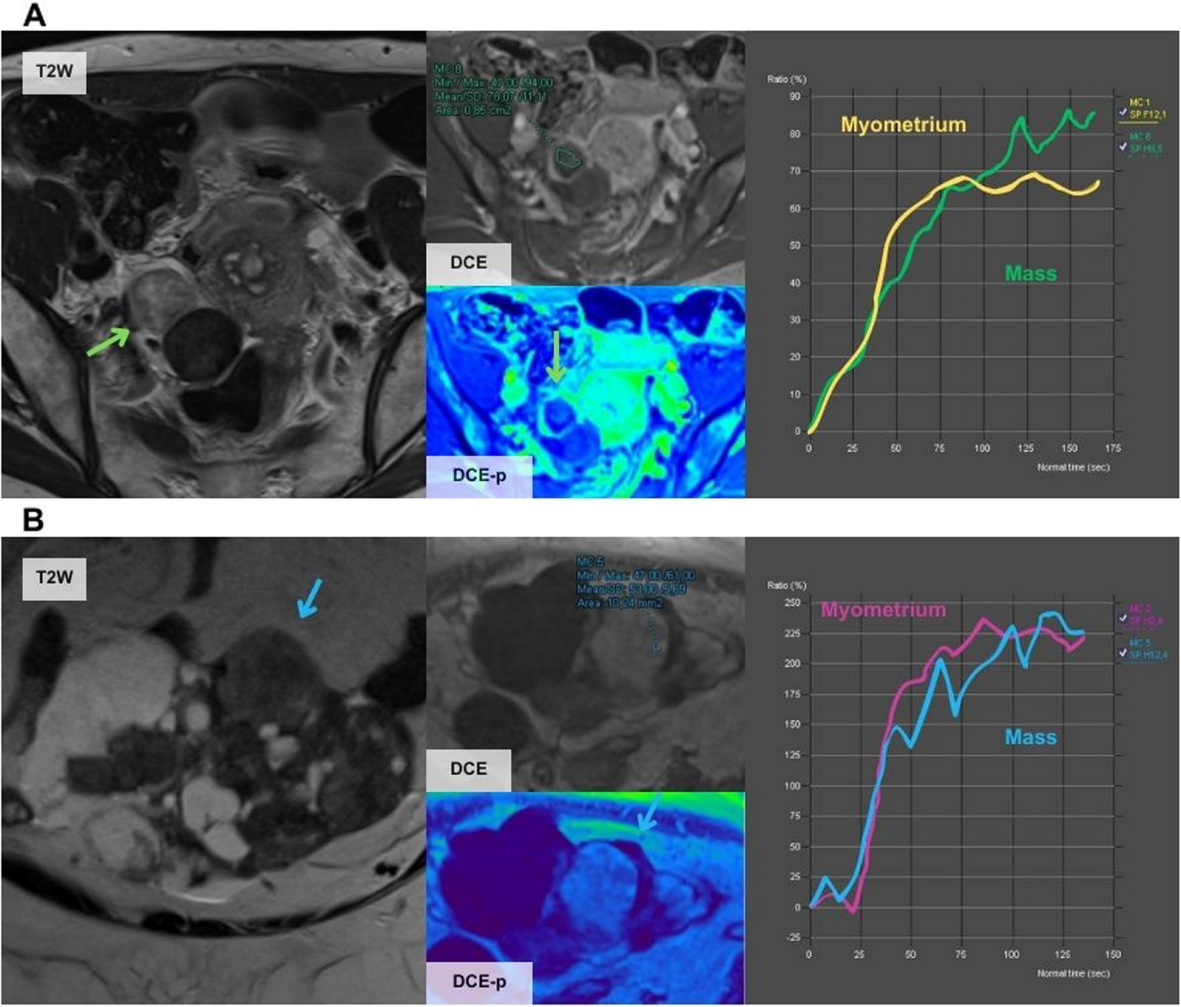
Erroneous classifications by the JR and the SR. **A** A 61-year-old woman presented an incidental right ovarian mass (green arrow) with a high-risk TIC classified as score 5 by both readers but was finally a fibrothecoma. As specified by O-RADS MRI guidelines, unenhanced sequences should be acquired before the contrast bolus injection. However, in this case, there was an error in the acquisition as there were no unenhanced sequences before the injection of the contrast bolus (in both TIC the contrast uptake started at the second 0), and this can distort the TIC results and lead to misinterpretation. In clinical practice, cases like this should be considered O-RADs 0 (incomplete or erroneous MRI technique). **B** A 56-year-old woman presented an incidental left ovarian mass (blue arrow) with a high-risk TIC that was classified as score 5 by both readers but was finally a luteinised fibrothecoma

**Fig. 4:**
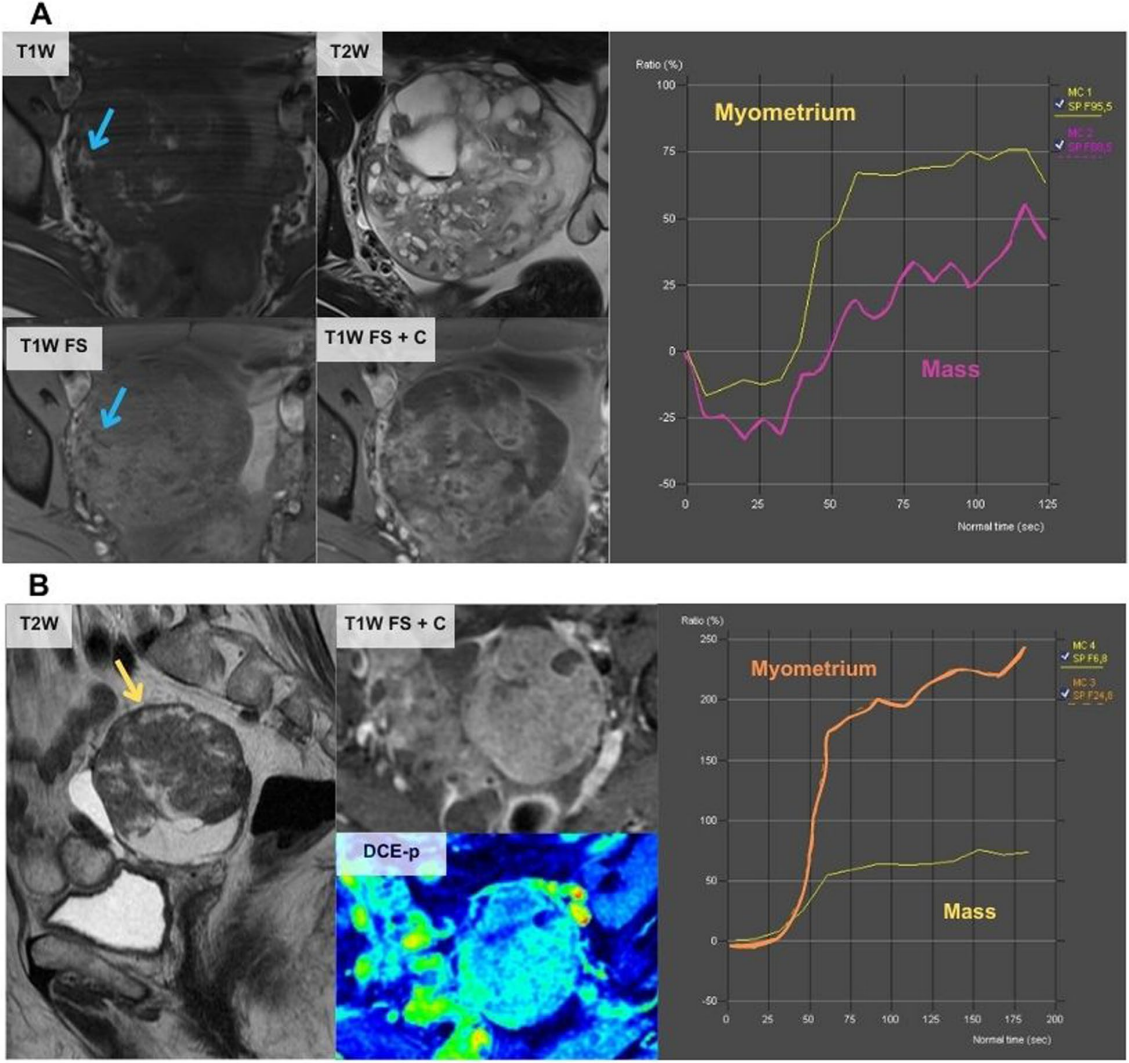
Errors by the JR due to misinterpretation of the classification. **A** A 31-year-old woman presented a right ovarian mass with macroscopic fat content (blue arrow) and a high amount of solid-enhancing tissue (T1W FS + C series). The mass does not have a Rokitansky nodule. It was classified as score 2 by the JR but the histological result showed an immature teratoma. Teratomas do not fit the classification well as they can have low, intermediate or high-risk TIC. In this case, the mass had an intermediate TIC. It is difficult to distinguish mature from immature teratoma and thus, it is stipulated that if they present a high amount of solid tissue, they should be classified with a score of 4. **B** A 79-year-old woman with a left ovarian mass (yellow arrow) with solid hyperenhancing tissue (T1W FS + C series). The TIC was interpreted as low risk by the JR and the mass was misclassified as score 3. Pathological analysis after surgery confirmed that it was a clear cell carcinoma arising from a cystadenoma. In this case, the TIC was an intermediate-risk curve as it had an initial increase lower than the myometrium, followed by a plateau

**Fig. 5:**
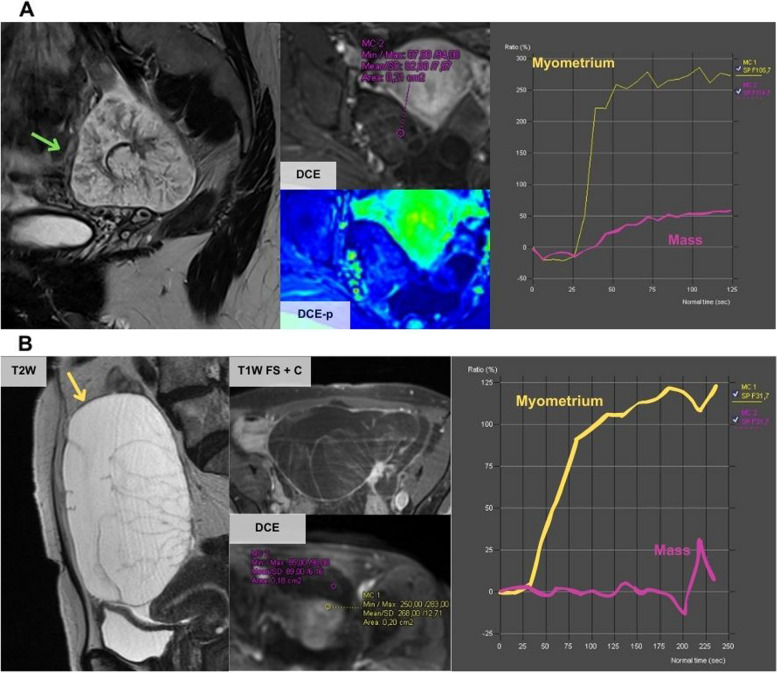
Errors by the JR, paradigmatic examples. **A** A 33-year-old woman with bilateral adnexal masses with a tree-like morphology (green arrow) and low contrast uptake shown by the TIC. This case was classified as score 3 by the JR as it was considered that it had a low-risk TIC. Pathological analysis after surgery confirmed that it was a borderline serous tumour. **B** A 67-year-old woman with a left ovarian mass that shows hyperintense T2w content and multiple thin septa (yellow arrow) typical of mucinous tumours. This mass was misclassified as score 3 by the JR as it was interpreted as having a low-risk TIC. The histological results showed metastasis of a mucinous appendix tumour

**Fig. 6:**
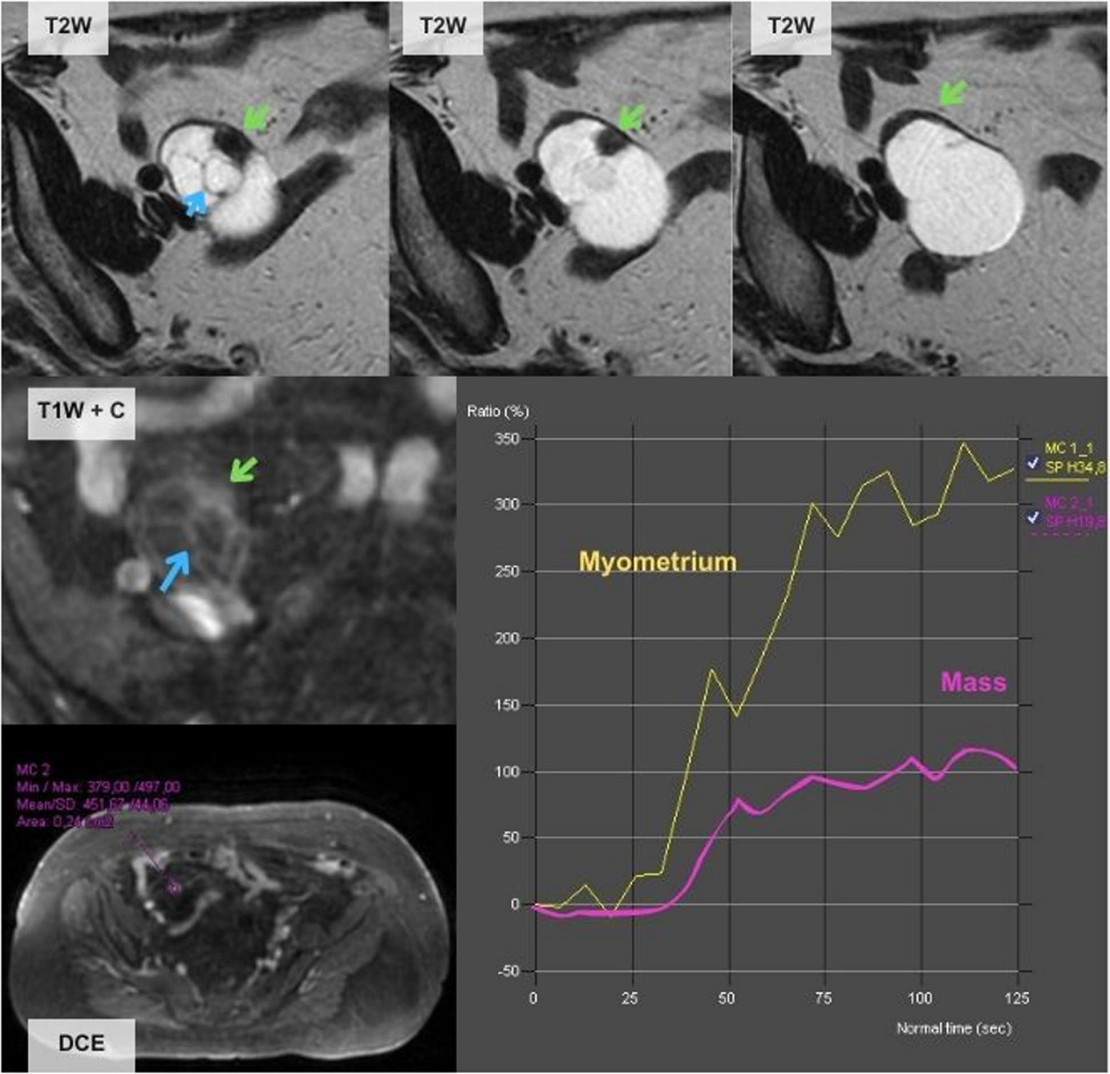
A 74-year-old woman with a right ovarian solid-cystic mass. This case was misclassified as score 4 by the JR considering it as having an intermediate-risk TIC. The postoperative pathological analysis revealed that it was a mucinous cystadenoma mixed with a Brenner tumour. In this case, the JR calculated the TIC out of the ovarian parenchyma surrounding the lesion (green arrow), but the true solid component was the thin septa (blue arrow) that corresponded to a score 3 as the SR perceived. As in this case, false positives may be due to errors performing the TIC

Overall, there were seven misclassified cases, five by the JR and two by both the JR and the SR. The cases misclassified by both radiologists (Fig. 3) corresponded to fibrothecomas that showed a solid component with a high-risk TIC. The other cases misclassified by the JR are shown in Figs. 4, 5, and 6 and correspond to a metastasis of a mucinous appendiceal tumour with a small solid component that was classified with a score of 3 because it had a low-risk TIC, a borderline serous tumour with a low-risk TIC that was classified with a score of 3, a solid-cystic mass classified as score 3 because of a misinterpretation of the TIC and was finally a clear cell carcinoma arising from a cystadenoma, a solid mass with macroscopic fat content that was classified as score 2 and was actually an immature teratoma, and finally, a solid-cystic mass with an intermediate-risk TIC classified as with a score of 4 that was a mucinous cystadenoma mixed with a benign Brenner tumour in the postoperative pathological analysis.

### Diagnostic performance

The diagnostic performance is summarised in Table 4.

**Table 4:** Diagnostic performance of O-RADS and interobserver agreement

	**Junior reader**		**Senior reader**	
	**%**	**95% CI**	**%**	**95% CI**
**Sensitivity**	96.1	89.0 to 98.9	97.4	90.9 to 99.3
**Specificity**	92	81.2 to 96.8	100	95.2 to 100
**PPV**	93.9	83.5 to 97.9	96.2	87.0 to 98.9
**NPV**	94.8	87.4 to 98	100	92.9 to 100
**Interobserver concordance (Kappa Index)**	92.3% (95% CI 85.9; 98.4)			

*CI* Confidence interval, *PPV* Positive predictive value, *NPV* Negative predictive value

The classification carried out by the SR obtained a sensitivity of 97.4% (95% CI: 90.9; 99.3), a specificity of 100% (95% CI: 95.2; 100.0), a PPV of 96.2% (95% CI: 87.0; 98.9), and a NPV of 100% (95% CI: 92.9; 100.0) for the prediction of malignancy.

The classification by the JR obtained a sensitivity of 96.1% (95% CI: 89.0; 98.9), a specificity of 92.0% (95% CI: 81.2; 96.8), a PPV of 93.9% (95% CI: 83.5; 97.9), and a NPV of 94.8% (95% CI: 87.4; 98.0) for the prediction of malignancy.

### Reproducibility

Considering the score range 2–3 as benign and the score range 4–5 as malignant, there was discordance in only 5 of the 126 studies (6%). The interobserver agreement between the JR and the SR was excellent, with a Kappa index of 0.92 (95% CI 0.86; 0.98).

## Discussion

Our results confirm that the O-RADS MRI effectively distinguishes benign adnexal masses from malignant regardless of the experience of the reader, with an accuracy of 98.4% achieved by the SR and 94.4% by the JR with high interobserver agreement (Kappa index of 0.92), consistent with recent literature [15, 18, 25–27].

Achieving an accurate O-RADS MRI classification starts with the correct MRI protocol. If this is not accomplished, the study is classified as O-RADS 0 [22]. One essential parameter is the assessment of solid tissue enhancement using a TIC comparing the kinetics of the mass-enhancing solid tissue to the myometrium, which was first described by Thomassin-Naggara et al*.* [28–30]. Specifically, the acquisition of the DCE series with two unenhanced sequences before the injection of the contrast bolus is important to avoid misinterpretation of the TIC as occurred in our series by both radiologists (Fig. 3). Another common mistake described in the literature and found in our study (Fig. 4B) is the differentiation between low and intermediate TIC [31, 32]. Rockall et al. published a great review of all the common technical mistakes that must be taken into account before designing an MRI protocol for the characterisation of ovarian masses [25]. Some recent studies also include semiquantitative parameters of the TIC to help avoid these mistakes [25, 33, 34].

Furthermore, O-RADS MRI scoring should not be used when there is suspicion of mass torsion, ectopic pregnancy, or pelviperitonitis regardless of whether these are suspected by clinicians or by image characteristics, as this can lead to false positives and false negatives [35, 36]. This occurred with only one of the 27 exclusions made in the present study due to the presence of acute pelvic symptoms. It is also important to note that if the mass presents enough specific radiological characteristics to achieve a certainty diagnosis, there is no need to use the O-RADS MRI classification [37, 38].

The number of cases classified as O-RADS 1 in our series was 31%—higher than what has been reported in the literature [18–20, 39–42]. More than 30% of erroneously considered ovarian masses by US in our series were uterine fibroids (Fig. 2). These can exhibit high-risk curves and ovarian fibromas almost always depict low-risk curves [43–45]. We believe that the increase in the number of O-RADS 1 in our series compared to the literature may be due to the inclusion of solid hypervascular masses identified by ultrasound that met simple rules features for category M, potentially leading to an increase in the inclusion of more subserosal fibroids and non-adnexal solid masses. The second most common extraovarian mass in our series was peritoneal inclusion cysts (9%) which characteristically appear surrounding the adnexa [46]. It is important to note that normal ovaries can show restricted diffusion and can depict high-risk TIC that can be mistaken for solid tissue, not only associated with peritoneal inclusion cysts but also in cystic ovarian masses (Fig. 6).

Fibromas were the most frequent solid benign mass in our series (Table 3) with 17 out of 77 benign lesions. They can show high Doppler vascularisation and are misclassified by US in up to 32% of cases [8]. The O-RADS MRI classification is extremely useful for these tumours as they show the typical “dark-dark” appearance (low signal in T2w and DWI b1000 sequences) that classifies them as score 2 [47]. Also, cystadenofibromas were a common finding in our series (7 out of 77 benign lesions) and were also well classified by the O-RADS MRI system, as they show characteristic imaging features, similar to fibromas but with a cystic component being classified as score 3 [48]. Fibromas and cystadenofibromas usually, but not always, depict low-risk curves. Therefore, solid or solid-cystic ovarian masses with marked hypointense T2w and low DWI in b 1000 in the solid tissue component should be considered as probably benign even if the middle risk curve is seen as in one of our mistaken classifications (Fig. 6).

Another challenging scenario and source of common mistakes in O-RADS MRI scoring are fat-containing lesions, both in our series (Fig. 4A) and as described in the literature [32]. The O-RADS MRI classification is ambiguous regarding the classification of fat-containing lesions and only suggests that lesions with a large amount of solid enhancing tissue should be classified as score 4 and those with less amount of solid tissue should be classified as score 2 [22]. Cheng et al. recently proposed the incorporation of fat characteristics as possible modifiers of the O-RADS MRI to improve classification performance [49].

Borderline tumours can be mistaken for low-risk lesions as occurred in our series (Fig. 5A) because they may have low contrast uptake. Nevertheless, these tumours usually have specific imaging features that can lead to diagnosis and must be classified as score 4 and treated as potentially malignant, due to uncertainty as to whether they are malignant or not [50]. Another type of lesion that is easy to misclassify is mucinous tumours (Fig. 5B), as they usually have a small solid component that can lead to false negatives in the elaboration of the TIC. It is also important to be aware of the possibility that these lesions may be either a primary mucinous tumour (benign or malignant) or a metastasis from a malignant extraovarian mucinous lesion [44].

A checklist (Table 5) can help to achieve the correct O-RADS score in every adnexal mass evaluated [44]. Other tools that can help are the MRI calculator available in the ACR website and the use of a structured imaging report [47, 51, 52].

**Table 5:**
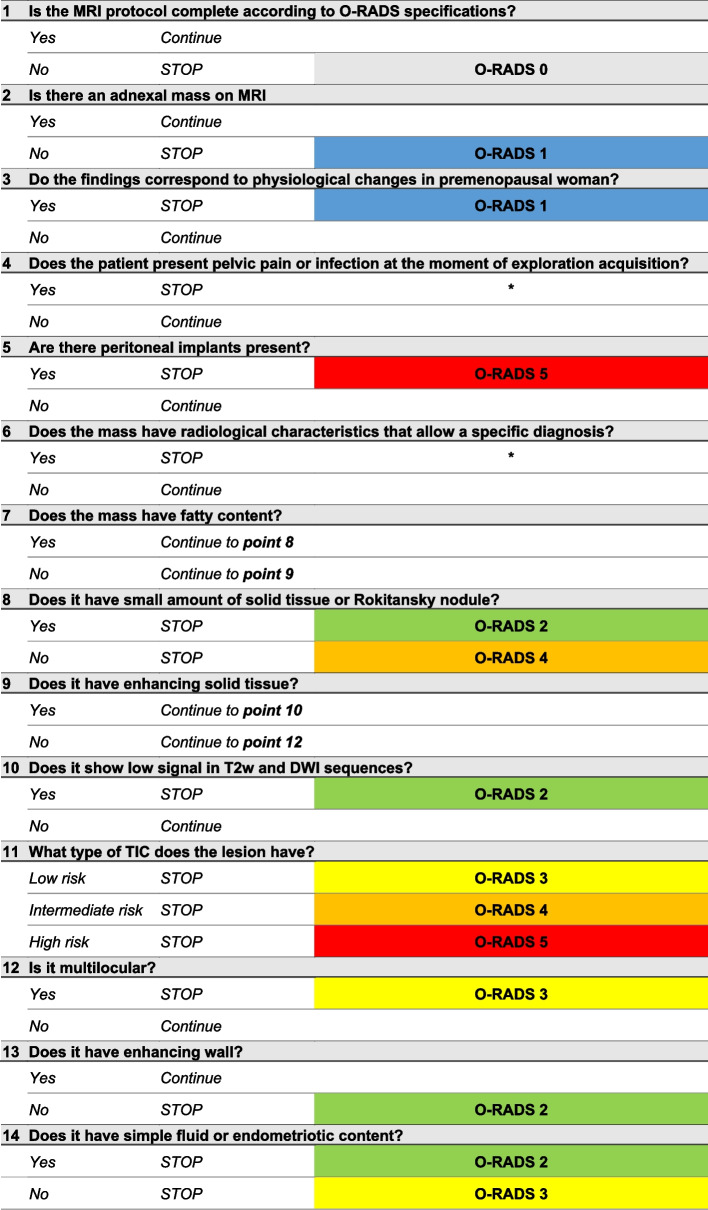
Checklist guide to score O-RADS MRI

^*^ Do not apply O-RADS classification if the diagnosis can be achieved with specific radiological characteristics or if torsion or pelvic inflammatory disease is suspected at the moment of the exploration  Note: The color of the shaded cells corresponds to each O-RADS MRI cattecory, grey for score zero, blue for score one, green for score two, yellow for score three, orange for score four and red for score five

Limitations of our study include being retrospective and single-institutional. The protocol used is not exactly the same in all our equipment (Table 1); the thickness in some series was 5 mm, which is greater than what is proposed in the literature; however, this makes the study closer to everyday working conditions and more reproducible [22, 25]. We did not include quantitative analysis of the MRI parameters, such as DWI, as we strictly followed the recommendations of the O-RADS MRI, but recent publications have shown the potential additional value of the use of apparent diffusion coefficient values to classify adnexal masses [53, 54]. These quantitative parameters could help to avoid upstaging solid masses with high-risk TIC, such as the fibrothecomas in our series.

In conclusion, indeterminate adnexal lesions remain an important workload in gynaecology departments. The O-RADS MRI classification can be used as a problem-solving tool, avoiding unnecessary invasive treatment. Its interpretation can be accurately achieved by specialists in gynaecological imaging as well as by inexperienced radiologists with the appropriate initial training.

## Data Availability

The data that support the findings of this study are available from the corresponding author, upon reasonable request.

## Abbreviations

ACR: American College of Radiology
CI: Confidence interval
DCE: Dynamic contrast enhancing sequence
DWI: Diffusion-weighted image
IOTA-SR: International Ovarian Tumour Analysis Simple Rules
JR: Junior radiologist
MRI: Magnetic resonance imaging
NPV: Negative predictive value
O-RADS: Ovarian-adnexal Reporting Data System
PPV: Positive predictive value
ROI: Region of interest
SR: Senior radiologist
TIC: Time-intensity curves
US: Ultrasound
